# Supplementary material for: Molecular Evolution of Histone Methylation Modification Families in the Plant Kingdom and Their Genome-Wide Analysis in Barley
Source: Int J Mol Sci. 2023 Apr 28;24(9):8043. doi: 10.3390/ijms24098043 (PMC10178440; doi:10.3390/ijms24098043)
Supplement: Supplementary file 1 [file ijms-24-08043-s001.zip › Supplementary Materials-Figures.pdf]

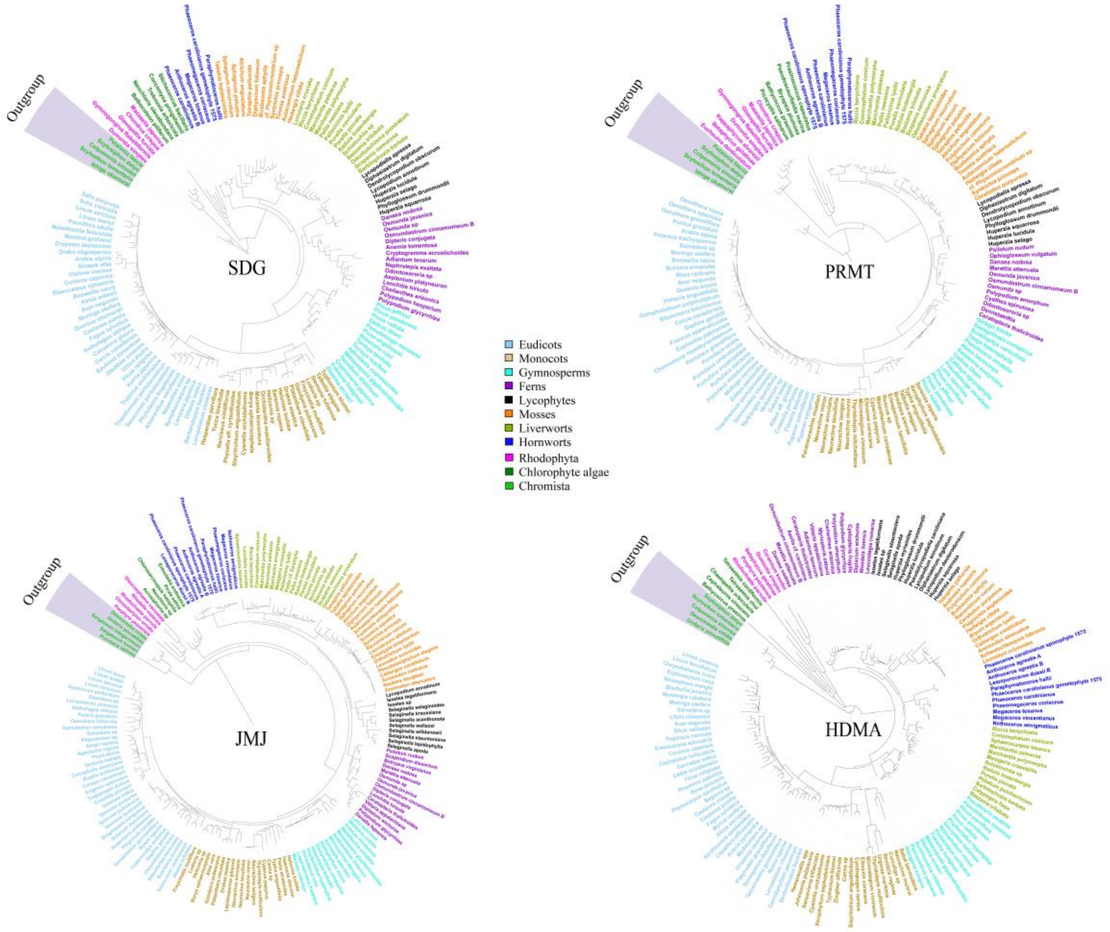

Figure S1. Evolutionary analysis of *HMT* (*SDG*, *PRMT*) and *HDM* (*JMJ*, *HDMA*) in representative species of the major lineage of green plants. *HMT*: Histone methyltransferases, *SDG*: SET domain group, *PRMT*: protein arginine methyltransferases, *HDM*: Histone demethylases, *HDMA*: SWIRM and C-terminal domain, *JMJ*: JmjC domain-containing proteins.

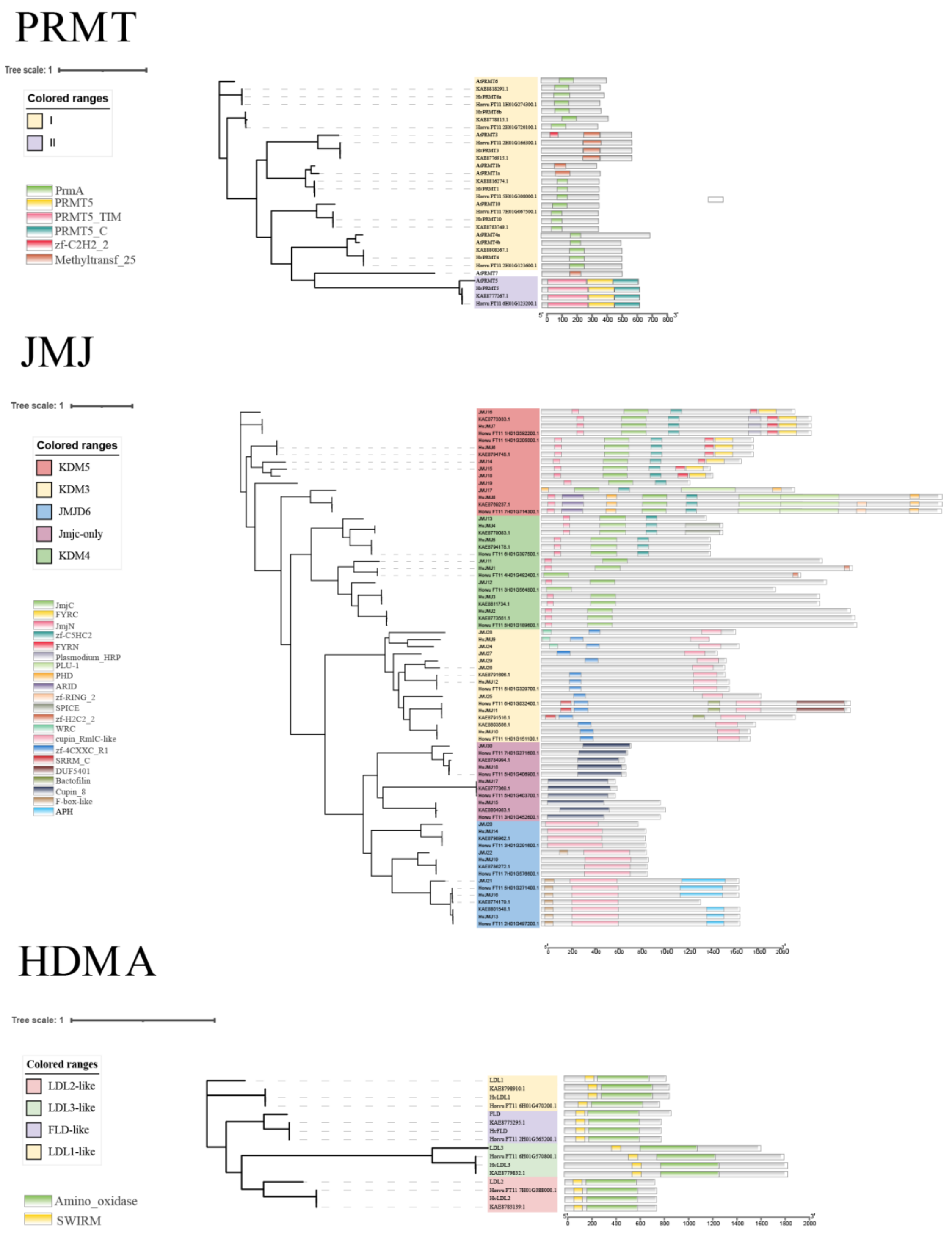

Figure S2. Phylogenetic and Conserved Domain Analysis of *PRMT*, *JMJ* and *HDMA*

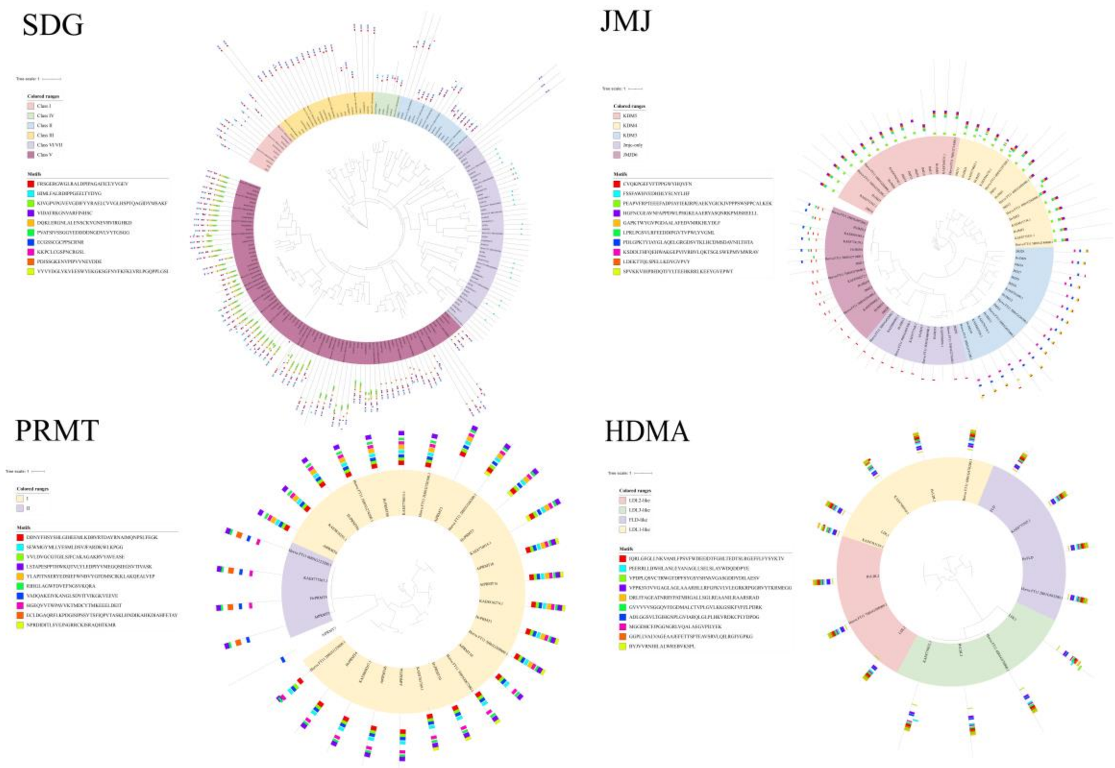

Figure S3. Motif distributions of *HvHMTs* and *HvHDMs*

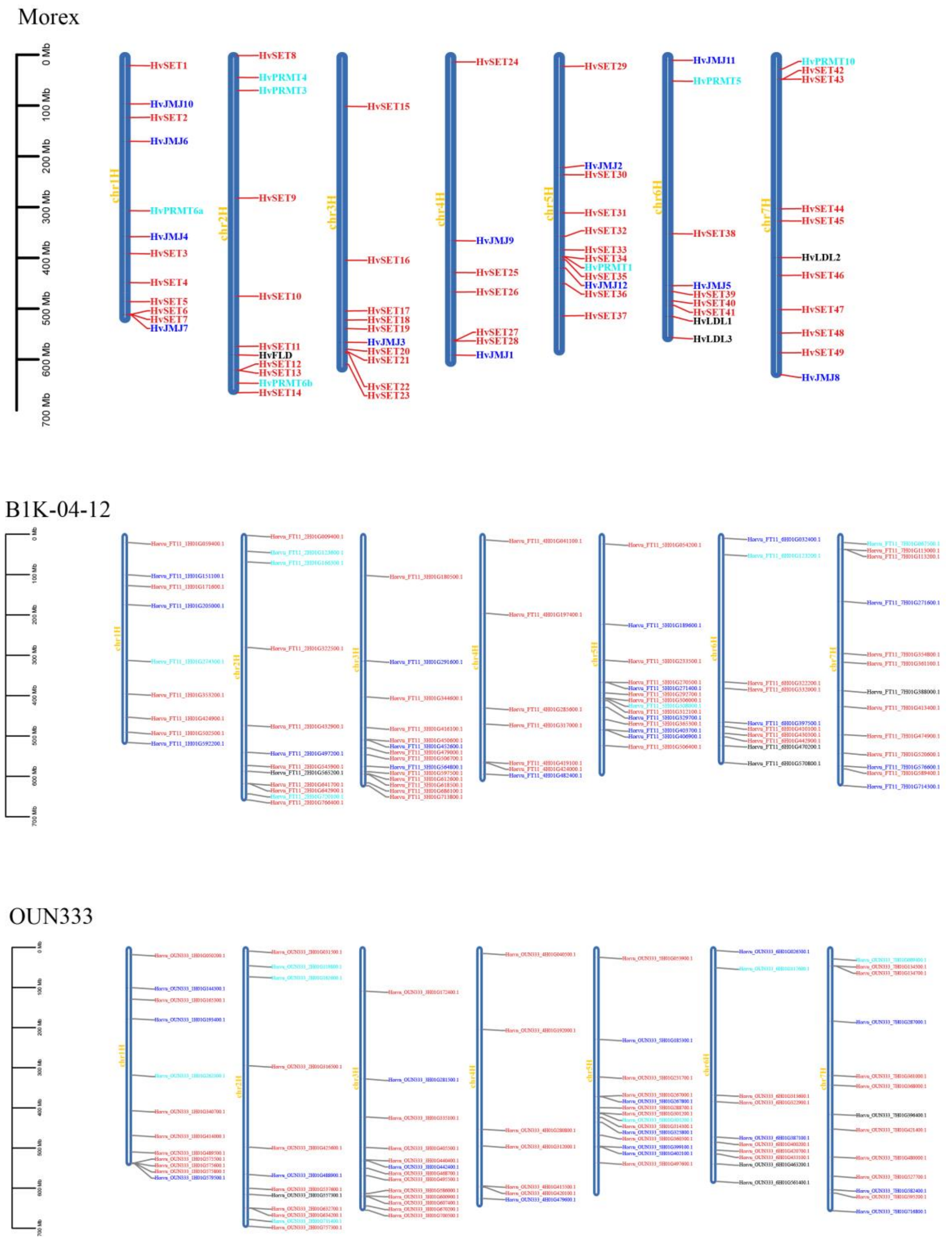

Figure S4. Chromosomal location analysis of *HMT* (*SDG*, *PRMT*) and *HDM* (*JMJ*, *HDMA*) families in different germplasms of barley. The red font is the *SDG* subfamily gene, the turquoise font is the *PRMT* subfamily gene, the blue font is the *JMJ* subfamily gene, and the black font is the *HDMA* subfamily gene
