# Supplementary figures and images for: Molecular Evolution of Histone Methylation Modification Families in the Plant Kingdom and Their Genome-Wide Analysis in Barley
Source: Int J Mol Sci. 2023 Apr 28;24(9):8043. doi: 10.3390/ijms24098043 (PMC10178440; doi:10.3390/ijms24098043)

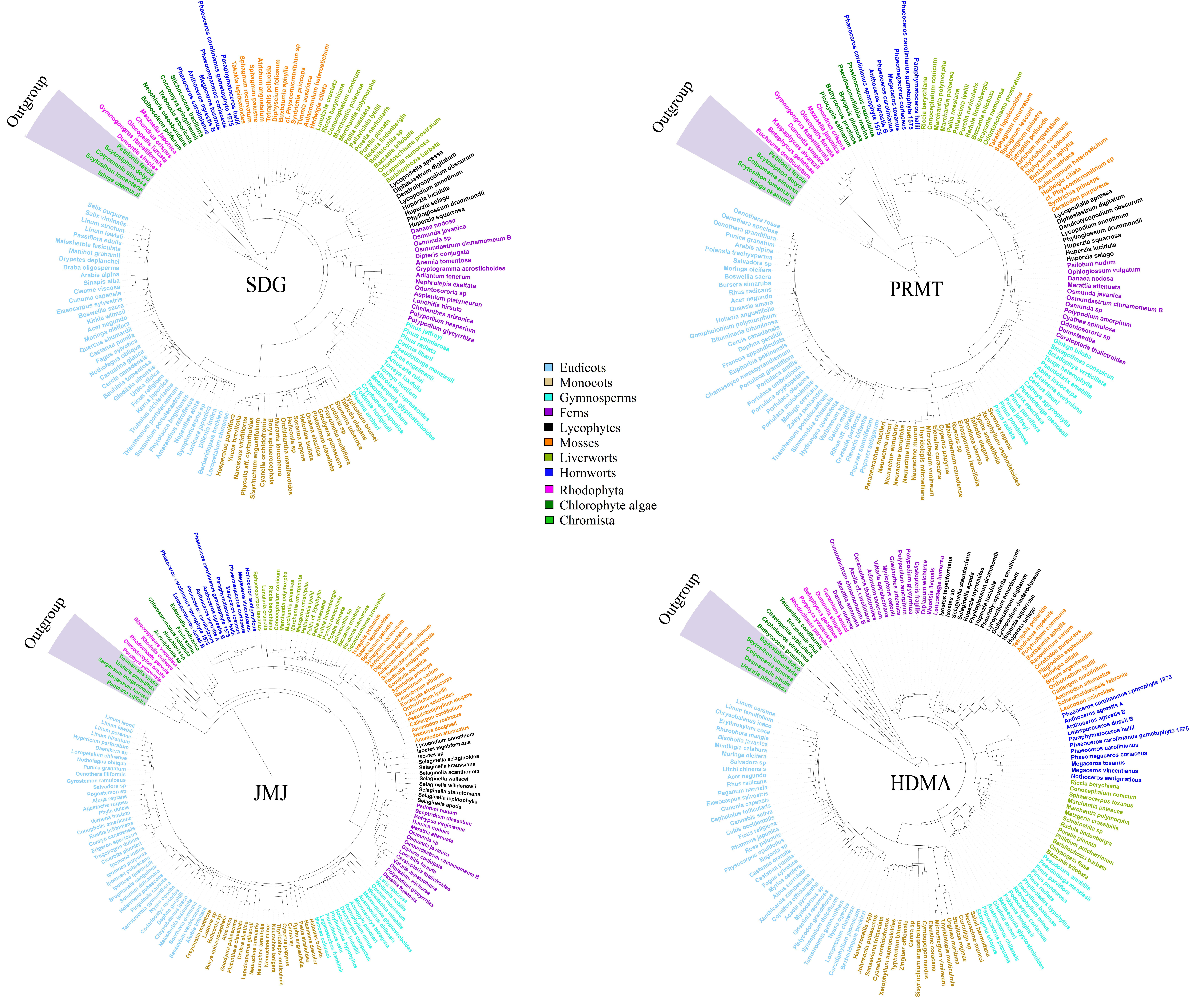

Supplement: Supplementary file 1 [file ijms-24-08043-s001.zip › FigureS1.jpg]

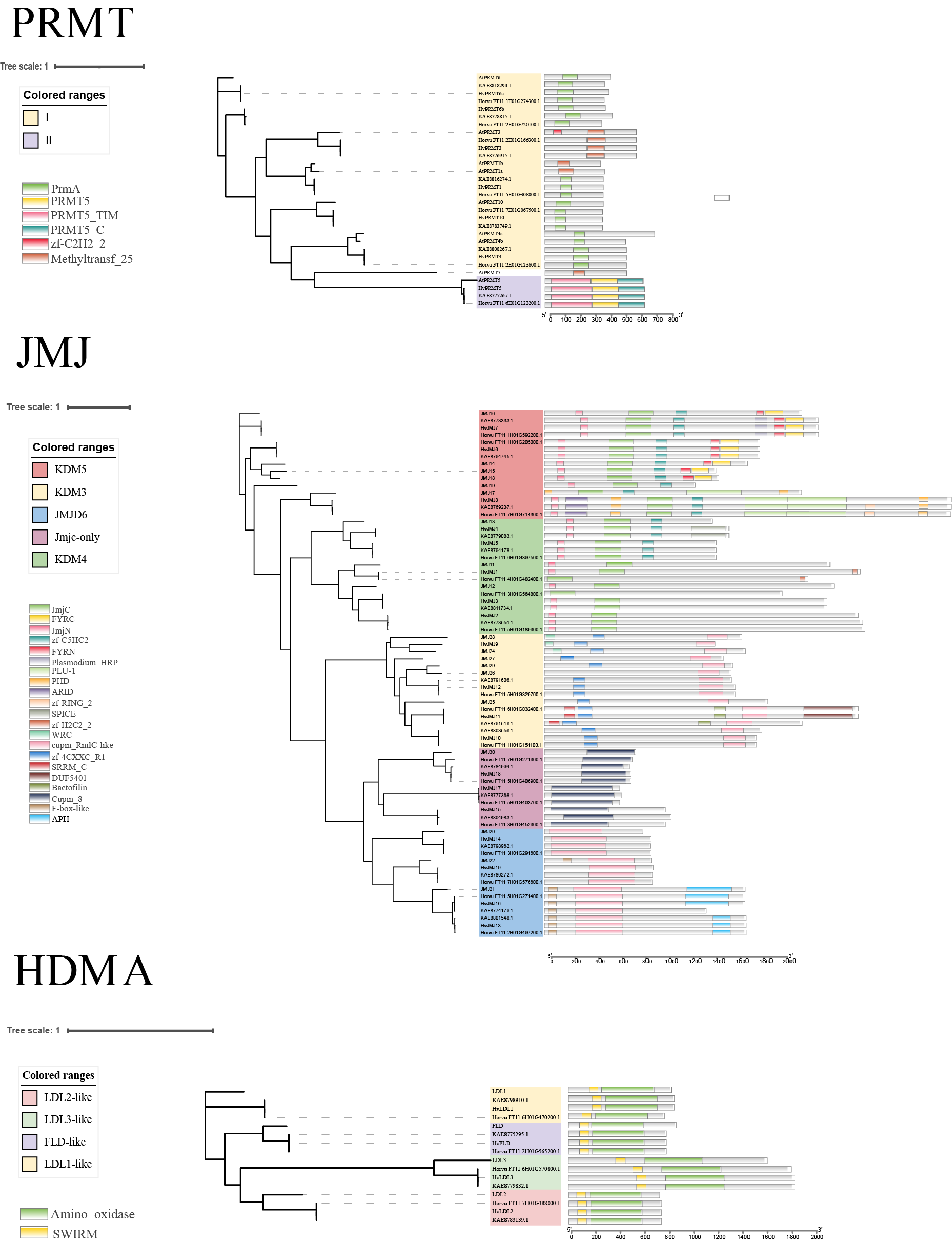

Supplement: Supplementary file 1 [file ijms-24-08043-s001.zip › FigureS2.tif]

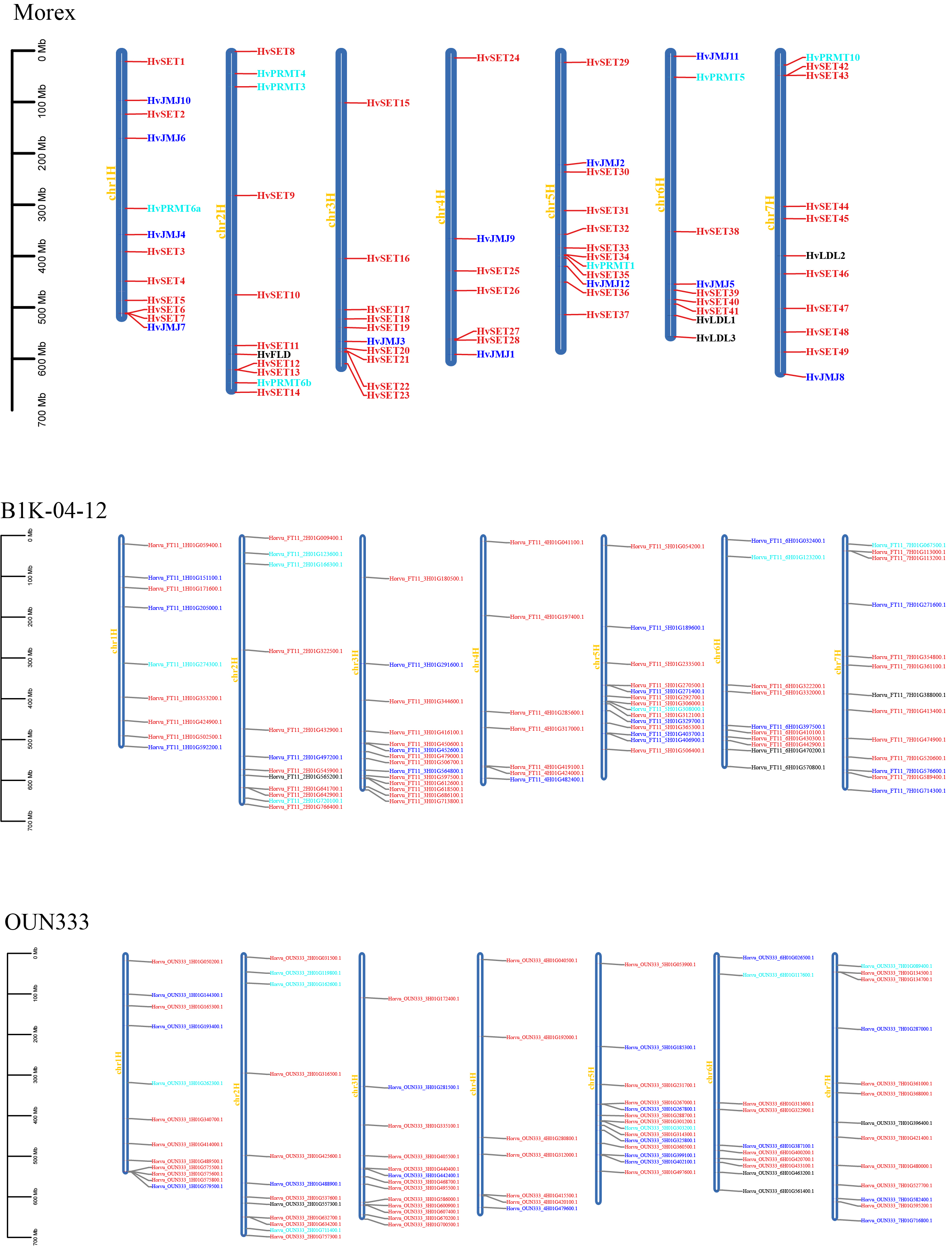

Supplement: Supplementary file 1 [file ijms-24-08043-s001.zip › FigureS4.jpg]
